# Intensity modulated radiotherapy for sinonasal malignancies with a focus on optic pathway preservation

**DOI:** 10.1186/1756-8722-6-4

**Published:** 2013-01-07

**Authors:** Alexander Chi, Nam P Nguyen, William Tse, Gill Sobremonte, Patrick Concannon, Angela Zhu

**Affiliations:** 1 Department of Radiation Oncology, West Virginia University, 1 Medical Center Dr. Morgantown, Morgantown, WV 26506, USA; 2 Department of Radiation Oncology, University of Arizona, Tucson, AZ, 85724, USA; 3 Department of Hematology Oncology, West Virginia University, Morgantown, WV, 26506, USA; 4 Department of Radiation Oncology, Michael E. Debakey VA Medical Center, Houston, TX, 77030, USA

**Keywords:** Intensity modulated radiotherapy, Sino-nasal malignancies, Optic toxicity, Blindness

## Abstract

**Purpose:**

To assess if intensity-modulated radiotherapy (IMRT) can possibly lead to improved local control and lower incidence of vision impairment/blindness in comparison to non-IMRT techniques when treating sinonasal malignancies; what is the most optimal dose constraints for the optic pathway; and the impact of different IMRT strategies on optic pathway sparing in this setting.

**Methods and materials:**

A literature search in the PubMed databases was conducted in July, 2012.

**Results:**

Clinical studies on IMRT and 2D/3D (2 dimensional/3 dimensional) RT for sinonasal malignancies suggest improved local control and lower incidence of severe vision impairment with IMRT in comparison to non-IMRT techniques. As observed in the non-IMRT studies, blindness due to disease progression may occur despite a lack of severe toxicity possibly due to the difficulty of controlling locally very advanced disease with a dose ≤ 70 Gy. Concurrent chemotherapy’s influence on the the risk of severe optic toxicity after radiotherapy is unclear. A maximum dose of ≤ 54 Gy with conventional fractionation to the optic pathway may decrease the risk of blindness. Increased magnitude of intensity modulation through increasing the number of segments, beams, and using a combination of coplanar and non-coplanar arrangements may help increase dose conformality and optic pathway sparing when IMRT is used.

**Conclusion:**

IMRT optimized with appropriate strategies may be the treatment of choice for the most optimal local control and optic pathway sparing when treating sinonasal malignancy.

## Background

Being less than 1% of all cancer, sinonasal malignancies frequently present in the locally advanced stage [1,2]. This poses a therapeutic challenge in the treatment planning and delivery of definitive or adjuvant radiotherapy due to the proximity of the primary tumors to critical normal structures, such as the optic nerves (ON) and chiasm (OC). Severe vision impairment and blindness are often encountered after therapeutic doses were delivered in the pre-IMRT era [3-19]. This was most commonly observed in the era of conventional radiotherapy (2D), when doses to the target volume and adjacent critical structures could not be well defined [3-9,14-19]. Due to the lack of the ability to deliver a highly conformal radiation dose, the clinical outcome in the 2 D era is also poor [3-9]. As an ideal approach to deliver a meaningful dose to the primary tumor or the postoperative tumor bed, and optimally preserve the optic pathway, which is often immediately adjacent to the planning target volume (PTV), intensity modulated radiotherapy (IMRT) has been utilized with excellent short term results [20,21]. Since the adaptation of IMRT into clinical practice, the clinical outcome of IMRT for sinonasal malignancies have been reported by many institutions with improved visual preservation [22-26]. However, there was no consensus on the dose limits for optimal visual preservation. Thus, we conduct this review to analyze if the incidence of vision impairment and blindness after IMRT is less frequent in comparison to conventional/three dimensional conformal radiotherapy (3D-CRT) when treating sinonasal malignancies and local tumor control follow IMRT when compared with non-IMRT techniques; the relationship of vision impairment and radiation dose to the optic pathway in this setting; and how various strategies of IMRT delivery may influence optic structure sparing.

## Methods and materials

This systematic review of literature was to investigate the clinical outcome and the incidence of vision impairment and blindness relative to the doses to the optic pathway after 2D RT, 3D-CRT, and IMRT for sinonasal malignancies in order to demonstrate if IMRT can potentially lower the incidence of vision impairment without any compromise of tumor control. In addition, we will explore the potential advantages & disadvantages of various IMRT delivery strategies for optic pathway sparing in this setting. A search based on PubMed electronic databases was conducted in July, 2012 to select studies outlining the following: clinical outcome of radiotherapy for sinonasal or paranasal malignancies; vision impairment and blindness after radiotherapy. The following terms were explored and used for each database search: sinonasal malignancies, paranasal sinus tumors, nasal cavity & ethmoid sinus tumors, vision impairment, optic neuropathy, optic retinopathy, blindness, radiotherapy, IMRT. For clinical reports, only the most complete and most recently published full length study reporting the clinical outcome and treatment related toxicity after 2D/3D RT, IMRT, or any combination of them for predominantly carcinomas from the sinonasal complex has been selected from any one institution. Two separate studies from one single institution were selected only if they described the outcome after RT delivered with two different techniques, or when one is reporting the outcome for exclusively one RT delivery approach and another is a comparison study of clinical outcome after RT delivered with various techniques. Studies describing the technical aspects of RT delivery were selected as long as the same investigation was not duplicated by the same group of authors. Conventional fractionation of 1.8 to 2 Gy per day is assumed unless otherwise indicated in this study.

## Results

Fifteen studies published between 1983 and 2009 [3-17], describing the clinical outcome and optic toxicity in detail after 2D or 3D RT; 2 studies published between 2008 & 2009 [18,19], reporting the clinical outcome & optic toxicity profile after RT delivered with a combination of 2D/3D RT & IMRT; 5 studies published between 2006 and 2012 [22-26], reporting the clinical outcome & optic toxicity after exclusively IMRT; and 3 studies published between 2007 and 2012 comparing the clinical outcome & incidence of optic toxicity after IMRT and non-IMRT techniques [27-29] were selected for the clinical portion of this review. Twelve studies describing the technical aspects of IMRT for paranasal malignancies were also selected for exploration of what would be the best approach to optimize optic pathway sparing [30-41].

### Local control & optic toxicity when IMRT is not used, or used in only a portion of patients

As shown in Table 1, treatment related blindness has been reported in all but one study which delivered a median dose of 54 Gy in 27 daily fractions when only 2D RT was delivered [3-9]. The local control from studies which reported them was 45.4%–62% (Table 1). The lowest local control was associated with a relatively high percentage of T4 disease and lower dose delivered with a hypofractionated course [6].

**Table 1:** Optic toxicity after 2D RT for sinonasal malignancies

**Ref.**	**#**	**Median follow up (mo)**	**Stage**	**RT**	**Presciption dose**	**Local control**	**Dose to the optic pathway**	**Vision impairment**	**Blindness**
Ogawa [3]^†§^	41	93	T_4_: 31.7%	2D	54 Gy/27 frx^ǂ^ (40–70 Gy)	59%	54 & 60 Gy^*^	7.3%	None
Tiwari [4]^†^	50	n/a	IV: 50%	2D	64–70 Gy/32–35 frx ± brachytherapy to 82 Gy^¶^	62%; 16/19 recurrences are stage III or IV	n/a	16% optic retinopathy & neuropathy	2% unilateral blindness
Jiang [5]^§^	219	n/a	n/a	2D	n/a	n/a	Retina: 58.3 Gy/29 frx^ǂ^.	Ipsilateral: 33.7%.	8.2% ipsilateral blindness due to optic neuropathy; bilateral blindness due to chiasm injury at 10 yrs:
							ON: 61.6 Gy/30 frx^ǂ^;	Retinopathy: 20% when 50–60 Gy received;	50–60 Gy: 8%;
							OC: 57.1 Gy/30 frx^ǂ^	Optic neuropathy: 2.3% when optic nerve received 44–60 Gy (56 Gy); 34% when optic nerve received 61–78 Gy at 10 yrs	61–76 Gy: 24%.
Logue [6]^§^	152	61	T_4_: 44%	2D	45-55 Gy/15–16 frx	45.4%	n/a	n/a	9.9% unilateral blindness, 2.0% bilateral blindness.
Sakai [7]^§^	171	~ 60	n/a	2D	50-70 Gy/25–35 frx	n/a	n/a	Ipsilateral: 74.6%	Ipsilateral: 63.7%
								Contralateral: 36.8%	Contralateral: 2.9%
Olmi [8]	69	n/a	T_3-4_: 88.4%	2D	42-72 Gy, 2 Gy daily or bid delivered with Co-60 or electrons	56%	n/a	n/a	13.6% (3/22) blindness (60 Gy/30 frx and 52 Gy/26 bid frx)
Nakissa [9]	30	n/a	n/a	2D	34-86 Gy, various fractionation	n/a	n/a	6.7% (after 65 Gy received)	6.7% who received > 68 Gy

*#* number of patients, *n/a* not available, *frx* fraction, ^*†*^postoperative only, ^*^associated with vision impairment or blindness, *ON* optic nerve, *OC* optic chiasm, ^*ǂ*^ median dose, ^*¶*^ average dose, *pt* patient, chemotherapy in a portion of pts^§^.

The local control reported in the four 3D studies was 56.4%–83%, while the percentage of locally advanced disease appear to be high when compared with that reported in the 2D studies [10-13] (Tables 1 and 2). The incidence of treatment related blindness appears to be lower in these studies. Only 1 such case was found 3 years after radiotherapy [12]. However, 41.2% patients experienced severe optic toxicity at 2 years after being treated with concurrently intra-arterial cisplatin and radiotherapy in one study [10]. This study also reported the best local control of 83% after a median follow up of 55.2 months.

**Table 2:** Optic toxicity after 3D RT for sinonasal malignancies

**Ref.**	**#**	**Median follow up (mo)**	**Stage**	**RT**	**Presciption dose**	**Local control**	**Dose to the optic pathway**	**Vision impairment**	**Blindness**
Homma [10]^§^	47	55.2	T_4_: 85.1%	3D	65 Gy/26 frx or 70 Gy/35 frx	83.0%	n/a	42.1% grade 3 or 4 optic toxicity at 2 years (*CTCAE v.3*); severe optic toxicity is 56% in patients with orbital invasion	None reported
Gabriele [11]^§^	31	42	T_4_: 38.7%	3D	60-68 Gy/30–34 frx	61.3%	ON:	None	2 due to disease progression, 1 due to cataract
						5 yr LC:	-Ipsilateral: 5.7-46.1 Gy		
						-Postoperative: 74%	-Contralateral: 3.8-25.6 Gy		
						-Definitive: 20%	OC: 11.3-36.2 Gy		
Pommier [12]^§^	40	19	T_3-4_: 55%	3D	Definitive: 63.5 Gy^¶^	80%	ON:	None	1 patient 3 years after RT due to vascular glaucoma
					Postoperative: 61.8 - 63.1 Gy^¶^		-Ipsilateral 48.1 Gy^¶^		
					2 Gy/frx		-Contralateral 22.0 Gy^¶^		
							OC: 43.5 Gy^¶^		
Roa [13]	39	54	IV: 53.8%	3D	Definitive: 68.4 Gy^ǂ^;	56.4%	n/a	1 retinal artery occlusion and 1 optic neuropathy after 68.4 Gy delivered.	None
					Postoperative: 55.8 -67.8 Gy^ǂ^				
					1.8-2 Gy/frx				

*#* number of patients, *n/a* not available, *frx* fraction, ^*†*^postoperative, ^*^associated with vision impairment or blindness, *ON* optic nerve, *OC* optic chiasm, ^*ǂ*^ median dose, ^*¶*^ average dose, *pt* patient, chemotherapy in a portion of pts^§^.

Severe vision impairment/blindness became more prevalent in studies that included patients who received 2D RT and those who were treated with 3D and/or IMRT [14-19] (Table 3). The local control in these studies was 35.9%–64.4%. The lowest local control (35.9%) was found in a study which only included patients with unresectable T4b disease treated to 70 Gy [18]. Otherwise, the local control was 58.5%–64.4% in these studies. It was 62% in the study which observed blindness due to disease progression only [16]. 56.67% of the patients had T4 disease on that study. A study that reported both 2D & 3D results demonstrated the actuarial rate of severe ophthalmological complications at 5 years to be 15% after 2D RT, but only 4% after 3D CRT even though this did not reach any statistical significance [15]. In the 2 studies which included 2D/3D RT, and IMRT, blindness is found 7 years after IMRT in one study when an area of the optic nerve received 77 Gy; and mostly after a definitive dose of 70 Gy/35 daily fractions was delivered in the other study [18,19].

**Table 3:** Optic toxicity after combined techniques for sinonasal malignancies

**Ref.**	**#**	**Median follow up (mo)**	**Stage**	**RT**	**Presciption dose**	**Local control**	**Dose to the optic pathway**	**Vision impairment**	**Blindness**
Jansen [14]^§^	68	66	T_4_: 63.2%	2D/3D	66 Gy/33 frx^ǂ^	64.4%	LON: 73.8	27.9%; 33.3% patients with orbital invasion experienced severe optic toxicity	22.1%
						5 yr LC:	Gy_2_^ǂ*^		
						Definitive: 47%	RON: 73.8 Gy_2_^ǂ*^		
						Postoperative: 65%	OC: 70 Gy_2_^ǂ*^		
Snyers [15]^§^	168	69	T_4_: 57.6%	2D/3D	64 Gy/32 frx^ǂ^	64%	1 case of unilateral blindness after both optic nerves received a maximum dose of 61 Gy	14% LENT SOMA grade 3–4 opthalmologic toxicity at 5 years (2D 15%, 3D 4%)	2.4% unilateral blindness
Porceddu [16]^§^	60	67	T_4_: 56.67%	2D/3D	50-70 Gy, 1.8-2 Gy/frx; 60 Gy/30 frx for 3D CRT	62%	ON/OC dose was limited to 54 Gy	n/a	3 due to disease progression
Blanco [17]^§^	106	60	T_4_: 55.7%	2D/3D	Definitive 61.7 Gy^¶^	58.5%	1 IMRT successfully spared optic nerves and chiasm with mean doses of 33.9 Gy & 38.9 Gy to those two structures	Optic neuropathy in 2 pts (1.9%) & optic retinopathy in 1 pt (0.9%); all led to blindness	2.8%
					Postoperative 60.9 Gy^¶^				
					Preoperative 55.7 Gy^¶^				
					Mostly 1.8-2 Gy/frx				
Hoppe [18]^§^	39	20	T4b: 100%	2D/3D: 69%, IMRT 31%	70 Gy/35 frx^ǂ^	35.9%	n/a	1 case of Optic neuropathy, 77 Gy to ON	1 due to optic neuropathy 7 years after IMRT
Mendenhall [19]	109	51.6	T4: 32%	2D/3D mostly	Preoperative: 55 Gy^ǂ^	5 yr LC: 63%	n/a	n/a	Definitive RT:
					Postoperative: 64.8 Gy^ǂ^	T1-3: 82%			-Ipsilateral: 25%
						T4: 50%			-Bilateral: 1.8%
					Definitive: 70 Gy^ǂ^	Definitive: 43%			Pre or post operative:
						Postoperative: 84%			-Ipsilateral: 7.5%
									-Bilateral: 1.9%

*#* number of patients, *n/a* not available, *frx* fraction, ^*†*^postoperative, ^*^associated with vision impairment or blindness, *ON* optic nerve, *OC* optic chiasm, ^*ǂ*^ median dose, ^¶^ average dose, *pt* patient, chemotherapy in a portion of pts^§^.

As shown in several studies, the incidence of vision impairment appears to correlate with the radiation dose received by the optic pathway [5,9,11-16]. The incidence of vision impairment appeared to be low when the optic pathway has received less than 60 Gy. As shown by Jiang *et al.* the 10 year incidence of optic neuropathy was 2.3% when the optic nerve (ON) received 44–60 Gy, which increased to 34% when the dose to the optic nerve was 61–78 Gy [5]. Optic chiasm injury, which led to bilateral blindness, increased from 8% to 24% when the OC dose increased from 50–60 Gy to 61–76 Gy [5]. In other studies, severe vision impairment/blindness was also more commonly found when radiation dose to the optic apparatus exceeded 60 Gy [9,13-15]. However, only 1 case of radiation related blindness was reported when the ON/OC dose was limited to < 54 Gy [11,12,16]. Among all seventeen studies, a low incidence of blindness due to disease progression was observed in two studies that reported outcome after 3D RT alone and 2D/3D RT [11,16]. Both studies did not report any treatment related vision impairment.

Chemotherapy was combined with RT (2.6%–100% of the patients in each study, mostly < 50%) predominantly in a sequential fashion in multiple studies [3,5-7,10-12,14-18]. In these studies, the effect of chemotherapy on RT-related vision impairment has not been well described other than the fact concurrent chemo-radiation as reported by Homma *et al.* has been associated with a high incidence of severe optic toxicity [10].

### Local control & optic toxicity after IMRT

Among the 5 studies [22-26] which reported the clinical outcome & toxicity profile after IMRT for sinonasal malignancies, no treatment related blindness was observed. Local control varied from 63.9% to 81% (Table 4). As shown in Table 4, no severe optic toxicity was reported when the dose to the ON/OC was kept to ≤ 54 Gy (conventional fractionation). However, because of the short follow-up, and the relatively small number of patients, it remains to be seen whether 54 Gy is the dose limit for optimal visual preservation. Late severe vision impairment of 1.4% was observed when < 5% of the optic pathway was allowed to receive ≥ 60 Gy [22]. Severe vision impairment due to tumor invasion is also reported by this group who treated the gross disease to 70 Gy. When compared with 3D or 2 D RT delivered at the same institution during an earlier era, three studies demonstrated noticeably improved clinical outcome and lower incidences of vision impairment and blindness after IMRT (Table 5).

**Table 4:** Optic toxicity after IMRT for sinonasal malignancies

**Ref.**	**#**	**Median follow up (mo)**	**Stage**	**Prescription dose**	**Local control**	**Dose to the optic pathway**	**Vision impairment**	**Blindness**
Madani [22]*CTCAE v2*	84	40	T4: 29%	70 Gy/35 frx	77.4%	ON/OC constraint: V_60_ <5%.	**Acute**: 1.4%	None
						Actual Dose to 2% vol^¶^:	**Late**:1.4% Grade 3 visual impairment related to IMRT in non-previously treated pts; 2.7% grade 3 impairment due to tumor invasion in both non-previously irradiated & re-irradiated pts	
						ON:		
						Ipsilateral 58.4 Gy; Contralateral 51.3 Gy		
						OC: 47.4 Gy		
Combs [23]	46	16	T4: 65%	PTV 60 Gy/30 frx, bst CTV 66 Gy/33 frx^ǂ^	81% at 2 yrs	ON/OC constraint: 54 Gy.	None	None
						RON: 37.9 Gy^ǂ^		
						LON: 37.4 Gy^ǂ^		
						OC: 25.3 Gy^ǂ^		
Daly [24]	36	51	T4: 69%	GTV 70 Gy/33 frx, CTV 58 Gy/33 frx^ǂ^	63.9%	ON/OC constraints: D_1%_ 54 Gy (ON), 45 Gy (OC).	N/a	None
						Actual D_max_:		
						OC: 52.3 Gy^¶^;		
						ON:		
						Ipsilateral 59.1 Gy^¶^		
						Contralateral 45.2 Gy^¶^		
Hoppe [25]^*^	37	28	T4: 55%	PTV1 70 Gy	72.9%	ON/OC constraint: < 54 Gy.	No RTOG grade 3–4 toxicity	None
				PTV2 60 Gy		Actual D_max_:		
				PTV3 54 Gy		ON		
				All in 33 frx		ipsilateral 53 Gy^ǂ^		
						contralateral 41 Gy^ǂ^		
						OC 50 Gy^ǂ^		
Wiegner [26]*CTCAE v3*	52	26.6	T4: 76%	High risk PTV 66 Gy/33 frx, 74.4 Gy, 1.2 Gy bid in 5 pts; SRS/SRT bst in 4 pts: 8 Gy x 1 frx or 5 Gy x 2 frx	75%	ON/OC constraint: 45 Gy, 63 Gy if treated with bid schedule.	1 grade 3 optic neuropathy related to herpes zoster infection, 1 grade 3 corneal ulcer	None
					Postoperative: 77%			
					Definitive: 60%			

#: number of patients, *PTV1: residual disease, PTV2: surgical bed/areas at high risk for microscopic involvement, PTV3: elective nodal regions; *n/a* not available; ^*ǂ*^ median dose; ^*¶*^ average dose; *ON* optic nerve; *OC* optic chiasm; *pts* patients.

**Table 5:** Optic sparing, IMRT vs. non-IMRT

**Ref.**	**Dose**	**Clinical outcome**	**Dose to optic structures**	**Vision impairment**
Dirix [27]	60-66 Gy/30–33 frx for both IMRT and 3D-CRT	LC: 76% vs. 67% at 2 yrs favoring IMRT (*p* = 0.06);	ON/OC constraint: ≤ 60 Gy for IMRT.	No vision impairment after IMRT
		DFS: 72% vs. 60% at 2 yrs favoring IMRT (*p* = 0.02)		15.8% radiation induced retinopathy after 3D CRT.
		OS: 89% vs. 73% at 2 yrs favoring IMRT (*p* = 0.07)		
Al-Mamgani [28]	IMRT(70%): 60–74 Gy	LC: 80% for IMRT & 64% for 3D CRT (*p* = 0.2)	Dmax^ǂ^ (*IMRT* vs. *3DCRT*) when 70 Gy/35 frx was prescribed:	Ocular toxicity: 32% after 3DCRT, 5% after IMRT (*p* < 0.0001)
	3DCRT(30%): 60–70 Gy		OC 47 Gy vs. 54 Gy	Blindness: 1 after IMRT, 3 after 3DCRT
			ON	Organ preservation 88% vs. 65% favoring IMRT (*p* = 0.01)
			-Ipsilateral 50 Gy vs. 56 Gy	
			-Contralateral 42 Gy vs. 48 Gy	
Chen [29]	2D 50–74 Gy	LC:	n/a	≥ RTOG grade 3 visual toxicity
	3D 50–73 Gy	2D: 55.9%		-2D 20%
	IMRT 66–72 Gy	3D: 67%		-3D 9%
		IMRT: 69.6%		-IMRT 0%
				Blindness is only observed after 2D RT only (5.1% of pts treated with 2D RT)

*ON* optic nerve, *OC* optic chiasm; ^*ǂ*^ median dose; *n/a* not available; *pt* patient.

Among IMRT-only studies, 16% & 56% of the patients in studies by Hoppe *et al.* & Wieger *et al.* respectively, have received chemotherapy as well [25,26]. In the IMRT vs. non-IMRT studies, 35.4% and 15% of the patients reported by Al-Mamgami *et al.* & Chen *et al.* received chemotherapy [28,29]. No clear effect of chemotherapy on vision impairment after RT was observed in these studies.

### Technique of IMRT delivery for the treatment of sinonasal malignancy

Among the 6 small studies [30-35] comparing IMRT and 2D/3D RT, IMRT was found to produce more conformal target volume coverage and/or improved sparing of optic structures when compared to non-IMRT techniques by some (Table 6). As shown by Claus *et al.* different IMRT strategies can lead to differences in PTV coverage and OAR sparing in the treatment of sinonasal malignancies [36]. Non-coplanar set up, increased number of beams & segments may lead to better target volume coverage and ON/OC sparing. Four studies further compared specific IMRT strategies [37-40]. Their findings are also described in Table 6. Although no differences between 5-beam coplanar & non-coplanar IMRT in OAR sparing or target volume was shown by Serre *et al.*[38], partially non-coplanar IMRT has been shown to improve target volume dose coverage and sparing of optic structures when compared with 9-beam coplanar IMRT with Wang *et al.*[37]. An advanced technique of IMRT delivery under image guidance, helical tomotherapy (HT), was shown to be mostly comparable to 7-beam non-coplanar IMRT in ON/OC sparing, but superior in target dose homogeneity by Sheng *et al.* when a preoperative dose of 50 Gy was prescribed [39]. On the contrary, it was shown to generate both superior target dose homogeneity and optic structure sparing when compared to 9–11 beam coplanar IMRT in delivering 70 Gy/35 fractions by Chen *et al.*[40].

**Table 6:** Techniques of IMRT delivery ± comparison with 2D/3D techniques

**Ref.**	**Techniques**	**Dose prescribed**	**ON/OC dose constraints**	**Findings**
Adams [30]	IMRT vs. 2D/3D RT	64 Gy/32 frx	Contra-lateral ON D_max_ ≤ 60 Gy	On average, IMRT decreased the D_max_ to the contralateral ON when compared with 2D & 3D RT(56.4 Gy vs. 65.7 Gy & 64.2 Gy), and minimized volume receiving <95% prescribed dose (8.5% vs. 15.1% & 14.7%)
Lee [31]	Static IMRT vs. 3D CRT	70 Gy/35 frx	D_max_ ≤ 60 Gy	IMRT improved PTV coverage by the dose prescribed in general (93.0 ± 2.2% vs. 89.0 ± 4.8%, *p* = 0.005), no benefit in OAR sparing.
O’Daniel [32]	Modulator IMRT vs. 3D CRT	60-66 Gy/30–33 frx	D_max_ ≤ 54 Gy	35% minimal transmission IMRT decreased Dmax to the ON/OC (*p* < 0.05), and is comparable to static IMRT in ON/OC sparing
Huang [33]	15 beam IMRT; Sequential tomotherapy (MIMic); 5-field 3D CRT; 3-field 2D RT	Minimal dose of 60 Gy to CTV & 70 Gy to GTV	D_max_ ≤ 54 Gy	IMRT achieved better GTV coverage & sparing of OC when compared to 2D & 3D RT
Mock [34]	Passive scanning PT; IMRT; 3D CRT; 2D RT	60-70 Gy to the PTV	≤ 50 Gy to ON/OC	Not significantly different, 3D CRT & IMRT achieved better OAR sparing than 2D RT. PT decreased OAR mean dose by > 60% when compared to 3D CRT & IMRT.
Pacholke [35]	6-7 beam coplanar IMRT; 3 field; 4 field	70.2 Gy/39 frx to PTV	Case dependent	IMRT did not demonstrate any clear advantage over conventional 4 field plans.
Claus [36]	4-11 beam IMRT, 5/9 beam setups were coplanar	70 Gy/35 frx	≤ 60 Gy	Increased beam & segment number and non-coplanar setup may lead to improved PTV dose coverage and improved OAR sparing in selected cases.
Wang [37]	9 beam coplanar IMRT; 5 beam non-coplanar/coplanar IMRT	63 Gy/35 frx to PTV	D_max_ < 50 Gy	Target dose better, mean dose to both ON & D_max_ to the contralateral ON was significantly lower with the 5 beam approach.
Serre [38]	5 beam coplanar IMRT; 5 beam non-coplanar IMRT	n/a	D_max_ < 55 Gy	No obvious difference in OAR sparing & target dose coverage was found
Sheng [39]	7 beam non-coplanar IMRT vs. HT	50 Gy/25 frx	n/a	Comparable PTV dose coverage & OAR sparing, although better sparing of ipsilateral eye & lens
Chen [40]	9-11 beam coplanar IMRT vs. HT	70 Gy/35 frx	D_max_: 54 Gy	HT reduced Dmax to OC, ipsilateral ON & retina; improved target dose homogeneity
Tsien [41]	9 beam coplanar IMRT vs. 3D CRT	70 Gy/35 frx	D_max_ ≤ 60 Gy	Clinical decision to spare the contralateral ON only can lead to improved PTV dose coverage and improved TCP when compared to bilateral ON sparing IMRT and 3D CRT.

*ON* optic nerve, *OC* optic chiasm, *PTV* planning target volume, *OAR* organs at risk, *PT* proton therapy, *HT* helical tomotherapy, *frx* fraction.

At last, the importance of incorporating clinical decision into the IMRT optimization process was demonstrated by Tsien *et al.*[41]. In their study to deliver 70 Gy, the decision to spare only the contralateral optic pathway led to noticeably improved PTV coverage when compared to 3D CRT, which may result in better tumor control when compared to 3D CRT, and IMRT plans which were optimized to spare bilateral optic pathways.

## Discussion

To our best knowledge, this is the first systematic review that investigates the clinical outcome and the level of optic pathway sparing after IMRT in comparison to 2D/3D RT for sinonasal malignancies. As described above, worse local control and high incidence of severe optic toxicity are mostly found after 2D RT (Tables 1, 2, 3 and 4). Improved local control and lower incidence of severe vision impairment was found after 3D CRT and IMRT, with IMRT being associated with the better local control and lower incidence of severe vision impairment in general (Tables 2, 4 and 5). As shown by Dirix *et al.* statistically significantly improved 2-year DFS and noticeably better toxicity profile was found following IMRT when compared with patients who were previous treated to the same doses with 3D CRT [27]. Although not statistically significant, improved local control in two other studies which compared IMRT and 3D and/or 2D RT were also seen, while one of them also demonstrated statistically significant improvement in optic organ preservation with IMRT over 3D CRT [28,29]. Further improvement in treatment outcome may lay in combining IMRT with newer class of systemic agents as those found in the treatment of other malignancies [42-45].

A few cases of blindness due to disease progression were reported in two studies [11,16]. No severe treatment related vision impairment was reported while a local control of approximately 60% was achieved in both studies. This may be due to the difficulty of controlling locally very advanced disease. These studies included 38.7% and 56.67% of T4 disease, yet none had patients receiving more than 70 Gy [11,16]. The local control rate after definitive RT delivered with mostly non-IMRT techniques [11,14,18,19] has been poor in general, especially for T4b disease even when a dose of 70 Gy was given [18]. Thus, a higher dose than previously used may need to be used when treating very locally advanced disease, with sacrificing the ipsilateral optic pathway being considered. On the other hand, superior conformal avoidance of the optic structures with IMRT may improve the outcome of definitive RT for sinonasal malignancies. This is evidenced by Wiegner *et al.* who observed a local control of only 60% after definitive IMRT delivering the conventionally accepted dose in a cohort of patients among whom 76% had T4 disease [26].

A high incidence of severe optic toxicity was observed following concurrent intra-arterial chemotherapy and 3D RT as shown by Homma *et al.* despite the excellent local control of 83% achieved in their study [10]. Thus, concurrent chemotherapy, such as intra-arterial cisplatin in this study, may potentially be a contributing factor to severe optic toxicity after radiotherapy for sinonasal malignancies. Such high rate of optic toxicity was not observed in other studies including patients who also received chemotherapy. Therefore, whether the addition of chemotherapy worsens optic toxicity following RT for sinonasal malignancies is unclear at this time.

IMRT can be challenging at times as no clear consensus exist on how to set the dose constraints for the optic structures (ON/OC). As previous described, a lower incidence of severe vision impairment has been reported in 2D/3D studies when the radiation dose to the optic pathway was kept to ≤ 60 Gy [3-19]. This finding was again evidenced in the IMRT studies. As an illustration, Madani *et al.* reported only 1.4% severe optic toxicity when < 5% of the optic pathway received more than 60 Gy [22]. The best chance of vision preservation and the avoidance of severe vision impairment appear to be associated with keeping the maximal radiation dose to the optic pathway to approximately 54 Gy or less [3-26]. This is also corroborated by the QUANTEC report on the radiation dose volume effect on the optic pathway [46]. However, it may be difficult to obtain if the tumor is close to the optic apparatus. Other confounding factors such as the volume of the optic apparatus receiving high radiation dose, the influence of chemotherapy and chronic conditions, such as, diabetes mellitus, warrants further investigation in the future [47]. Altered fractionation has not been shown to be associated with worse optic toxicity profiles when compared with other studies in which conventional fractionation is used [5,6,8,9,25]. This is especially true when the fractional dose was only slightly above 2 Gy [5,25]. pt]?>Thus, the dose volume effects of altered fractionation schedules with > 2 Gy per fraction will need to be further characterized in the future since many studies were conducted in the pre-3D era when dose to different structures cannot be accurately estimated.

Only small studies on a limited number of patients have conducted to compare IMRT with non-IMRT techniques, and various IMRT strategies [30-41]. Thus, no clear demonstration of the superiority of IMRT over 2D/3D techniques can be made based on these dosimetric studies. However, increased magnitude of intensity modulation through increasing the number of segments, beams, and using a combination of coplanar and non-coplanar arrangements may help increase dose conformality and optic pathway sparing when IMRT is used to treat sinonasal malignancies. This is suggested in studies which demonstrated HT’s superiority over linac-based coplanar IMRT in target dose homogeneity and optic structure sparing; but comparable optic pathway sparing when HT and non-coplanar IMRT were compared [39,40]. HT’s delivers image guided IMRT with intensity modulation through 51 equally spaced angles [48]. One important component of IMRT optimization to treat sinonasal malignancies is the incorporation of clinical decision into the optimization process. As shown by Tsien *et al.* decision to spare the contralateral ON only may lead to improved IMRT plan quality and potentially better tumor control in certain cases [41].

## Conclusion

Dose conformality to the target volume and conformal avoidance of the organs at risk achieved through IMRT may provide better local control and less optic toxicity compared to conventional radiotherapy technique. A dose of 54 Gy or less delivered with conventional fractionation to the optic apparatus may provide optimal visual preservation if the tumor is not close to these critical structures. Further prospective studies with long-term follow-up should be conducted to assess how best to preserve vision and improve local control.

## Competing interests

The authors declare that they have no competing interests.

## Authors’ contribution

AC and AZ originated the idea and AC and NP conducted the design of this review. AC, NP, WT, GS, CP, and AZ conducted the data gathering and the writing of this manuscript. All authors approved this final version.
